# Experimentally Assessed Reactive Aggression in Borderline Personality Disorder

**DOI:** 10.1371/journal.pone.0166737

**Published:** 2016-11-16

**Authors:** Olga Kogan-Goloborodko, Elisabeth Brügmann, Jonathan Repple, Ute Habel, Benjamin Clemens

**Affiliations:** 1 Department of Psychiatry, Psychotherapy and Psychosomatics, Medical School, RWTH Aachen University, Aachen, Germany; 2 Department of Psychiatry, University of Münster, Münster, Germany; 3 JARA – BRAIN Institute I, Aachen, Germany; Boston Children's Hospital / Harvard Medical School, UNITED STATES

## Abstract

Approximately 73% of patients suffering from Borderline personality disorder (BPD) exhibit aggressive behaviour, which severely hinders therapeutic work and clinical improvement. Because the underlying mechanisms of aggression in BPD are not yet completely understood, additional research in this domain has a high clinical and scientific relevance. We employed a modified version of the Taylor Aggression Paradigm (mTAP), in order to examine for the first time whether this task can be used to differentiate between BPD patients and healthy controls with regard to reactive aggression. In the mTAP, the amount of money subtracted by a virtual opponent was categorized into ‘low’ (10–20 cents) and ‘high’ (80–100 cents) provocations, enabling us to compare how much money BPD patients and healthy controls subtracted (i.e., how aggressively participants responded) following high and low provocation trials. Our results showed that, compared to healthy controls, BPD patients showed higher overall aggression, higher aggression after high provocation trials, as well as a larger difference between high and low provocation trials. This finding was corroborated by a neuropsychological assessment, demonstrating higher levels of aggression and impulsivity in BPD patients. Interestingly, reactive aggression in the mTAP was positively correlated with symptom severity and impulsivity in BPD patients. We suggest that the mTAP provides a valuable tool allowing psychiatrists to quantify reactive aggression in BPD. Therefore, clinicians and researchers might consider this task, as a short experimental measure of reactive aggression, either in future studies or to aid diagnostic assessment during clinical practice.

## Introduction

Aggression and ensuing acts of violence are ubiquitous phenomena with substantial costs to our society, documented daily in the media. A recent World Health Organization report provided a 1-year worldwide estimate of 1.43 million people dying from either self-inflicted or interpersonal violence (excluding armed conflict), with a much larger number of nonfatal victims of violence. Importantly, most acts of violence were unplanned, occurring as a result of reactive aggression. In contrast to premeditated aggression, reactive aggression is associated with increased levels of autonomic arousal and precipitation by provocation related to negative emotions such as anger [1]. Regarding the propensity to aggressive behavior, there are multiple factors interacting with each other. These factors include genetic [2–5] and environmental [6, 7] influences, as well as dysfunctional brain activity and/or anatomical brain damage, predominately localised to prefrontal and limbic structures [8, 9].

Most likely, interplay between all of the aforementioned factors is at work when an individual exhibits some form of aggression. Heightened aggression can be found in patients suffering from Borderline personality disorder (BPD), which is one of the most functionally debilitating and costly psychiatric conditions. Regarding the lifetime prevalence in the general population, different studies have postulated estimates between 1.4% and 5.9%, with higher prevalence in females, individuals younger than 30, and in individuals coming from lower income surroundings [10–13]. The most significant and common symptoms of BPD are high levels of impulsivity and aggression, as well as emotional dysregulation [14, 15]. Approximately 73% of BPD patients present some form of aggressive behaviour directed towards others [14]. However, extreme forms of aggression involving violent acts such as killing, raping or severely assaulting people with lethal consequences occur rather rarely in BPD patients. Furthermore, BPD patients exhibit an increased risk for victimisation by personal assault, child maltreatment, and psychological aggression. Interestingly, neuroimaging studies of BPD patients indicate that aberrant neural activity in the dorsolateral prefrontal cortex (DLPFC) and the limbic system [16–19]. It is hypothesized that decreased inhibition of the limbic system by the DLPFC and other prefrontal structures represents a crucial neurobiological mechanism responsible for increased aggression and impulsivity in BPD [15].

Although pharmacological treatment for aggression in BPD exists, there are drawbacks related to side effects, non-responding and incompliance. Importantly, heightened aggression in BPD does not only result in individual suffering, but also in substantial complications during clinical routine. Therefore, additional research on the origins of aggression in BPD patients has a high clinical and scientific relevance. Within the field of clinical psychology, a popular option for behavioural measurements of reactive aggression, which is typical for BPD, is the Taylor Aggression Paradigm [20]. Previous studies already demonstrated satisfactory construct, internal, discriminant as well as external validity for this task [21, 22]. However, there is no study measuring the level of reactive aggression of BPD patients using the TAP, and therefore we decided to assess the TAP in this group of patients. We used a modified version of the TAP (mTAP), during which participants are provoked by a virtual opponent, who subtracts money from them in the context of a competitive reaction time game. Aggressive behaviour in reaction to provocation is measured by recording the level of “punishment” in the form of money deduction participants administer to their opponent.

The present study compared reactive aggression of BPD patients with a group of age-matched healthy controls, with the hypothesis that BPD patients will show increased reactive aggression in the context of the mTAP. As it is common in the literature to speak of ‘reactive aggression’ in the context of the mTAP, we followed this terminology for the present study. Of course, this form of aggressive behavior does not perfectly resemble real-world, physical and verbal aggression during everyday life. But in scientific laboratories and in empirical research in general, the sort of aggression elicited by the mTAP represents one of the most accurate and realistic forms of aggression we can investigate systematically. Nevertheless, several other complex issues, such as response to unfairness, strategic thinking, social desirability, and problem solving capabilities might also influence the behavior of participants during the mTAP. But we agree with the consensus in the literature [20–22], that the most influential factor being measured with the mTAP is reactive aggression itself. Reactive aggression in this context might then be best described as a form of human aggressive behavior that is unplanned and impulsive, occurring as a response to feelings of anger, fear or need for retaliation (e.g., punish your opponent in the mTAP, by taking more money from him after being provoked in the previous trial).

An important goal of the present study was to provide an additional external validation for this laboratory measure of aggression: although the (m)TAP has been employed repeatedly in studies of aggression, so far it remains unknown whether the mTAP is able to differentiate between psychiatric patients with heightened aggression (e.g. BPD patients) and healthy controls. If we are able to show that the mTAP reliably differentiates between patients and controls, this task might be employed in future studies and clinical settings in order to support diagnostic assessment, providing an additional measure to characterize the level of reactive aggression in BPD patients. In agreement with previous studies, we expected higher aggression levels in the group of BPD patients, due to affective dysregulation and increased impulsivity. Furthermore, we used several psychometric questionnaires to provide additional data on differences in personality and demographic characteristics between BPD patients and healthy controls.

## Methods

### 2.1. Participants

20 female patients (mean age = 29.55 years; SD = 10.66) with a diagnosis of BPD were recruited from the Department of Psychiatry, Psychotherapy and Psychosomatics of the RWTH Aachen University. A clinician confirmed the BPD diagnosis, according to the criteria of the DMS-IV, and the patients’ ability to participate in the study. Some of the patients took medications at the time of study, including: antidepressants (n = 13), sedatives (n = 5) and mood stabilizers (n = 8). As commonly found in studies with BPD patients, most patients did not only suffer from BPD. Comorbid diagnoses included depression (n = 7), anxiety disorder (n = 1), substance-abuse (n = 6), and eating disorders (n = 4). Patients with a diagnosis of an acute substance withdrawal syndrome were excluded.

The control group of 19 healthy women (mean age = 27.58 years; SD = 9.6) was recruited via public advertisements. A semi-structured clinical interview [23] was used to verify the absence of any psychiatric diagnosis in the control group. Furthermore, healthy controls were excluded if they reported any other medical condition. The authors assert that all procedures contributing to this work were approved by the Ethics Committee of the Medical Faculty of the RWTH Aachen University (EK 185/11) and comply with the Helsinki Declaration of 1975, as revised in 2008. For the present study, all participants provided written informed consent and received compensatory payment.

### 2.2. Procedures

All participants were told that the aim of the study was to evaluate concentration and reaction times. Prior to the experiment, participants completed a series of neuropsychological tests and questionnaires. This test battery included the MWT-B (German estimation of crystallized intelligence) [24], the TMT (Trail making test) [25], assessing executive functions and attention, the RWT [26], for verbal fluency and executive functioning, the WMS-R (Wechsler Memory Scale—Revised) [27], measuring verbal short-term memory, and the PPI-R (Psychopathic Personality Inventory-Revised) [28], evaluating various features of personality. Additionally, all BPD patients completed the BPI (Borderline personality inventory) [29], assessing symptom severity and the diagnostic criteria of BPD.

Upon arrival, participants were introduced to their opponent, which was an instructed associate (always portrayed by the same person) of the experimenter. Then both were instructed about the competitive RT task, with the participant playing in one room and the opponent playing in an adjacent room. Participants were told that if they responded faster than the opponent to the appearance of a visual cue, they would win the trial and receive 50 cents as a reward. Additionally, if they won, participants had to subtract money from the opponent (between 10 and 100 cents). Accordingly, if the opponent won a trial, he also subtracted money from the participant. Unbeknownst to the participant the trial outcome of the RT task and the amount of money the virtual opponent subtracted were predefined and identical for all participants. Each trial started with a visual analogue scale (VAS) that was used to indicate how much money the participant wanted to subtract from the opponent in case he won the following trial. The VAS could be adjusted from 10 to 100 cents in steps of 10 cents. Adjustment time was 4 s, and afterwards a white frame was displayed, indicating the start of the RT task. During the RT task, the participant was instructed to press the response button with the right index finger as fast as possible whenever she saw the visual cue. This cue appeared between 1 and 3 s after the RT task had begun. Following the RT task the participant received visual feedback (happy or sad smiley, shown for 4 s) regarding the trial outcome and the amount of money lost or gained. There were 151 trials in total. A detailed debriefing was conducted after all participants completed the task.

Several questionnaires were collected immediately before and after the mTAP. The STAXI (State Trait Anger Expression Inventory) [30] was used to assess the expression and control of anger, the PANAS (Positive and Negative Affect Schedule) [31] was employed to assess positive and negative affect, and the ESR (Emotional Self Rating) [32] assessed the emotional state of the participants. After the mTAP, the following questionnaires were obtained: the AQ (Buss–Perry Aggression Questionnaire) [33], assessing the overall aggression level, the RPQ (Reactive–Proactive Aggression Questionnaire) [34], differentiating between proactive and reactive aggression and the BIS-11 (Barratt Impulsiveness Scale) [35], for the behavioural construct of impulsivity.

### 2.3. Statistical Analysis

Using the SPSS 20.0 software package (IBM, Armonk, NY, USA), we employed two-sample t-tests to compare the means of the demographic data and the neuropsychological data. The same methods, as well as an analyses of variance (ANOVA) model, were used to compare the means of the aggression parameters of mTAP between the BPD and healthy control group. The amount of money subtracted by the virtual opponent was categorized into ‘low’ (10–20 cents) and ‘high’ (80–100 cents) provocations, and we compared how much money BPD patients and healthy controls subtracted following those high and low provocation trials (difference high vs. low provocation). Additionally, overall aggression (average amount of money subtracted across all trials) and aggression (i.e. amount of money subtracted) after low provocation as well as aggression after high provocation were compared between the two groups. The mTAP data were also Pearson correlated with the total scores of the BPI and the BIS-11. Additionally, the pre and post measurements, namely STAXI, PANAS and ESR, were analysed using repeated measures ANOVA with ‘session’ as within-subject factor and ‘group’ (patients vs. controls) as between-subject factor. The significance level for all statistical comparisons was set to p < 0.05.

## Results

### 3.1. Demographics and neuropsychological testing

There were no significant differences with regard to age (p = 0.549, t(37) = -0.606) or the level of crystalized intelligence (p = 0.082, t(36) = 1.790), between BPD patients and healthy controls. However, years of education differed significantly (p < 0.001, t(36) = 4.569), with less education reported by BPD patients. The TMT provided the following results: whereas no significant differences were found for the TMT-A (p = 0.056, t(35) = -1.974), which assesses basic attentional functions and processing speed, BPD patients exhibited significantly slower reaction times than healthy controls in the TMT-B (p = 0.001, t(36) = -3.810), measuring cognitive flexibility and executive functioning. In the PPI, the BPD group achieved higher scores in blame externalization (p < 0.001, t(36) = -6.625) and scored higher on the sub-scale for carefree aimlessness (p = 0.001, t(36) = -3.553). In contrast, healthy controls had a significantly higher stress immunity (p < 0.001, t(36) = 6.019). The remaining sub-scales of the PPI did not show a significant difference. Furthermore, the BPD group reported significantly higher levels of impulsivity on the BIS-11 (p < 0.001, t(37) = -6.022). BPD patients also reported higher aggression, as measured with the AQ (p < 0.001, t = -7.019) and the RPQ (p < 0.001, t(35) = -4.846). An overview of all demographic and neuropsychological data is presented in Table 1.

**Table 1:** Demographic and neuropsychological data.

	BPD (n = 20)	HC (n = 19)	p-value
Age	29.55 (± 10.66)	27.58 (± 9.60)	.549
Years of education	9.68 (± 2.43)	12.42 (± .95)	**< .001**
IQ (MWT-B)	94.21 (± 9.00)	99.32 (± 8.57)	.082
Borderline symptomatology (BPI)	24.83 (± 9.10)	-	-
Processing speed (TMT-A)	23.94 (± 7.33)	19.82 (± 5.23)	.056
Cognitive flexibility (TMT-B)	53.89 (± 20.27)	34.14 (± 10.00)	**.001**
Aggression (AQ) Total Score	88.60 (± 19.79)	53.26 (± 9.71)	**< .001**
Reactive aggression (RPQ) Total Score	17.20 (±8.84)	6.29 (± 3.00)	**< .001**
Impulsivity (BIS-11) Total Score	74.45 (± 8.22)	59.42 (± 7.31)	**< .001**
Blame externalization (PPI)	41.16 (± 9.11)	23.89 (± 6.79)	**< .001**
Carefree aimlessness (PPI)	35.89 (± 7.92)	27.84 (± 5.91)	**.001**
Stress immunity (PPI)	28.05 (± 7.41)	42.21 (± 7.083)	**< .001**
Aggression overall (mTAP)	64.44 (± 17.21)	42.92 (± 32.82)	**.017**
Difference high-low provocation (mTAP)	16.76 (± 12.84)	7.62 (± 11.34)	**.024**
High provocation (mTAP)	73.16 (±18.59)	49.34 (±36.53)	**.014**
Low provocation (mTAP)	56.92 (±18.45)	41.73 (± 33.63)	0.86

(AQ = Buss–Perry Aggression Questionnaire; BIS-11: Barratt Impulsiveness Scale; BPD = Borderline personality disorder; BPI = Borderline personality inventory; HC = Healthy controls; IQ = Intelligence quotient; mTAP: Modified Taylor Aggression Paradigm; MWT-B = Mehrfachwahl Wortschatztest; TMT-A/B = Trail making test; PPI = Psychopathic Personality Inventory-Revised; RPQ = Reactive–Proactive Aggression Questionnaire)

### 3.2. Modified Taylor Aggression Paradigm

**Fig 1:**
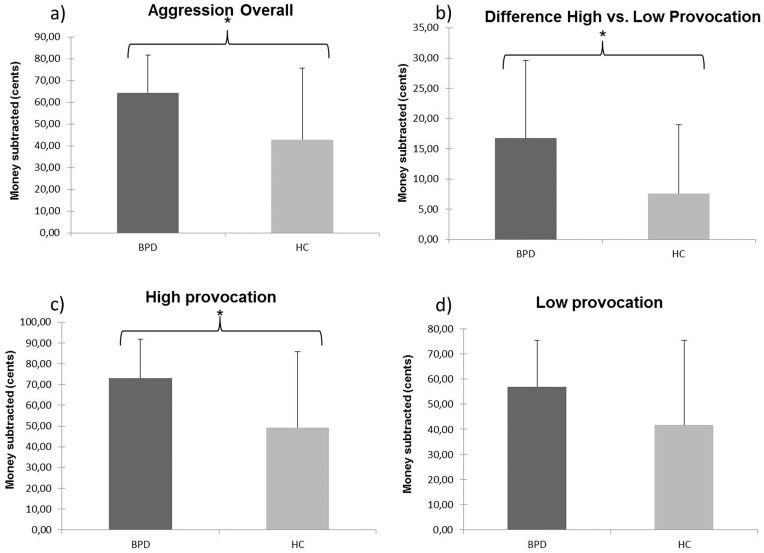
Differences in reactive aggression between BPD patients and healthy controls. Visualized are data from the modified Taylor Aggression Paradigm (mTAP), comparing the results of patients with Borderline personality disorder (BPD) and healthy controls (HC). Error bars represent standard deviation (SD). (A) The BPD group showed significantly higher aggression overall, which is represented by the money subtracted from the opponents. (B) The patients also had a significantly larger difference of subtractions when comparing trials following high provocation vs. trials following low provocation. (C) A significantly higher aggression following high provocation trials for BPD patients was also observed. (D) BPD patients also showed a numerically, but not significantly, higher aggression following low provocation trials.

BPD patients (64.442 ± 17.217) showed significantly higher overall aggression (p = 0.014, t(37) = -2.584), as compared to healthy controls (42.91 ± 32.817). The difference between subtractions after high and low provocation trials was also significantly larger (p = 0.024, t(37) = -2.351) for BPD patients (16.755 ± 12.841) as compared to healthy controls (7.617 ± 11.341). In order to test whether the two groups differed with respect to high and low provocation trials, we conducted a 2x2 ANOVA, with ‘group’ (BPD patients vs. healthy controls) as between-subjects factor and ‘level of provocation’ (high vs. low) as within-subjects factor. Both the main effect for group (F_1,37_ = 5.032, p = 0.031) and the main effect for level of provocation (F_1,37_ = 35.473, p = < 0.001), as well as the interaction (F_1,37_ = 4.638, p = 0.038), were significant. Post-hoc examination of subtractions after low and high provocation trials revealed that BPD patients (73.162 ± 18.590) subtracted significantly more money from their virtual opponents than healthy controls (49.343 ± 36.529) following high provocation trials (p = 0.014, t(37) = -2.586), but not following low provocation trials (p = 0.086, t(37) = -1.761). Data from the mTAP is visualized in Fig 1, and summarized in Table 1.

### 3.3. Pre vs. post measurements

**Fig 2:**
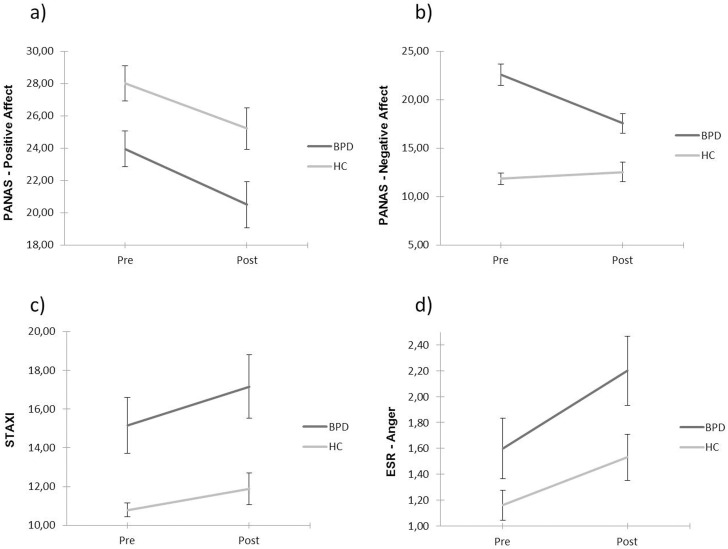
Emotional and effective changes following the modified Taylor Aggression Paradigm. Here, data from the State Trait Anger Expression Inventory (STAXI), Positive and Negative Affect Schedule (PANAS) and the subscale anger of the Emotional Self Rating (ESR) is shown. Pre and Post represent the time points immediately before and after the mTAP. Error bars represent standard error of the mean (SEM).

Concerning positive affect (PANAS), repeated-measures ANOVA revealed a significant main effect of group (F_1,37_ = 9.328, p = 0.004) and a significant main effect of session (F_1,37_ = 9.692, p = 0.004). Whereas both groups reported less positive affect after the mTAP, BPD patients already started with a lower level of positive affect. Concerning negative affect as assessed by the PANAS, we found a significant session by group interaction (F_1,37_ = 21.098, p < 0.001), as well as significant main effects for group (F_1,37_ = 42.00, p < 0.001) and session (F_1,37_ = 12.162, p = 0.001). After the mTAP, a well-marked decrease in negative affect for the BPD group was apparent, whereas negative affect for healthy controls remained stable. Furthermore, a significant main effect of group was found for the STAXI (F_1,36_ = 11.494, p = 0.002), with the BPD group reporting a higher level of anger before and after the mTAP. For the subscale anger of the ESR, we also found a significant main effect of group (F_1,37_ = 5.660, p = 0.023), and a main effect of session (F_1,37_ = 7.160, p = 0.011). A pronounced increase of anger in the ESR could be observed for both groups after the mTAP, and BPD patients reported significantly more anger only after the mTAP. The aforementioned data from the PANAS, STAXI and ESR scale ‘anger’ is visualized in Fig 2.

The subscale fear of the ESR showed a significant session by group interaction (F_1,37_ = 17.742, p < 0.001), as well as a significant main effect of session (F_1,37_ = 17.742, p < 0.001) and group (F_1,37_ = 15,269, p < 0.001). While BPD patients reported significantly less fear after the mTAP, healthy controls reported low, constant levels of fear before and after the task. In the subscale sadness, there was a significant main effect of group (F_1,37_ = 7.357, p = 0.010), showing the BPD group at a constantly higher sadness level than the control group. Concerning the happiness subscale of the ESR, there was a significant main effect of session (F_1,37_ = 6.610, p = 0.014), showing a steep decrease of happiness after the mTAP in both groups. No significant group differences were found for the ESR sub-scales surprise and disgust.

### 3.4. Correlations

**Fig 3:**
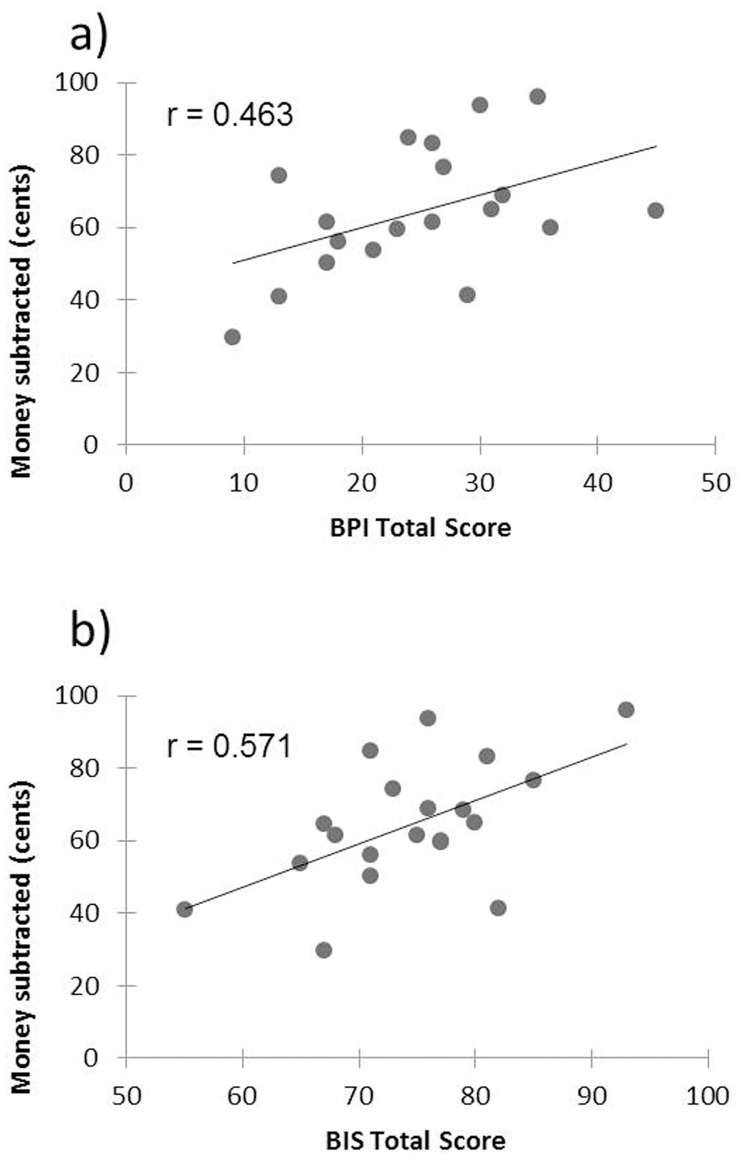
Relationship between reactive aggression and Borderline symptom severity and impulsivity. For the group of BPD patients, the figure visualizes the correlation (Pearson’s r) between reactive aggression (i.e., mean amount of money subtracted from the opponent during the mTAP), and the total scores of the Borderline personality inventory (BPI) and the Barratt Impulsiveness Scale (BIS-11). We found a significant positive correlation between reactive aggression and the BPI total score, which represents an overall indicator of BPD symptom severity, and between reactive aggression and the BIS-11 total score, which provides a reliable measure of impulsivity.

A significant positive correlation between overall aggression in the mTAP and the BPI total score (r = 0.463, p = 0.046), and between overall aggression in the mTAP and the BIS-11 total score (r = 0.571, p = 0.009) was observed. A graphic illustration of these correlations is provided in Fig 3.

## Discussion

In the present study we employed the mTAP, in order to examine for the first time whether this task can be used to differentiate between BPD patients and healthy controls with regard to reactive aggression. Our hypothesis regarding differences in aggressive behaviour was confirmed: compared to healthy controls, BPD patients showed higher overall aggression, higher aggression after high provocation trials, as well as a larger difference between high and low provocation trials. As stated previously [14, 15], we suggest that these findings indicate that BPD patients indeed have problems with emotion and affect regulation, specifically regarding suppression of negative emotions such as anger: the failure to regulate and inhibit negative feelings and emotions, which were most likely induced by high-provocation trials in the mTAP, led BPD patients to respond more aggressively and subtract more money from their opponents, making them more vulnerable and susceptible to high levels of provocation. As there was no difference in aggression following low-provocation trials, we suggest that BPD patients might be particularly prone to act overly aggressive in situations involving exceptionally high incidents of provocation. This might also have crucial implications for clinical practice, as it might be more effective for therapeutic settings to focus on such high-provocation scenarios.

Our results go in line with previous findings by Dougherty and colleagues (1999), who showed how severe negative affect in women with BPD is reflected in objective measures of impulsivity [36]. The major difference between this previous and the present study is that the task used by Dougherty and colleagues (1999) to provoke participants was designed to specifically assess impulsive behavior, and more specifically the inability to delay gratification. The mTAP on the other hand was specifically designed and optimised to assess reactive aggression. An important motivation to specifically employ this task was the fact that despite recent findings showing that 73% of BPD patients engage in violent, aggressive behaviour directed towards others [14], there are no specific tasks, laboratory measures or diagnostic tools suitable to asses this overly aggressive behavior directed towards others. As we have shown that the mTAP is indeed able to differentiate between BPD patients and healthy controls, clinicians and researchers could consider including the mTAP as a measure of reactive aggression in future studies.

Furthermore, the results of the pre and post measurements, assessing changes in anger, affect and emotionality, demonstrated that the mTAP indeed influenced affective and emotional processing in BPD patients and healthy controls. Together with the results from the mTAP itself, this indicates that BPD patients were indeed more affected by the provoking task than healthy controls. When assessing the relationship between experimentally induced aggression, assessed in mTAP, and different questionnaires, we found a significant positive correlation between overall aggression and the BPI total score, which assess BPD symptom severity. We also found a significant positive correlation between overall aggression and the BIS-11 total score. Taken together, we suggest that these results provide a preliminary validation for the mTAP as a laboratory measure, and potentially also diagnostic tool, for reactive aggression in BPD. The pre and post measurements also confirmed some typical behavioural features of BPD. Especially the decrease of negative affect and fear is very interesting and could be explained with an inadequately high tension and exceptionally high levels of expectation before the start of the experiment, which are followed by a feeling of relief after the experiment is finished. This interpretation is supported by a statement of Conklin and colleagues (2006), suggesting that BPD patients are characterized by more negative and less positive affect, and affect dysregulation, showing an inadequate reaction to stressful stimuli typically observed in BPD patients [37]. Herpertz and Bertsch (2014) described BPD patients as persons who are not able to distinguish between their own emotions and those of others as they were hypersensitive to their social environment [38]. Furthermore, BPD patients experience significant problems in managing and venting their emotions, especially anxiety and anger, which could have also influenced the results obtained in the present study.

In another study, Mancke and colleagues (2015) presented a multidimensional model of aggression in BPD patients [39]. This model explains the formation of aggression from the perspective of the bio-behavioural dimensions of affective dysregulation, impulsivity, threat hypersensitivity, and empathic functioning. The results obtained from the mTAP presented here seem to specifically support the aspect of increased impulsivity in the development of aggression. In addition to affective dysregulation, we would classify increased impulsivity as the most influential factor causing aggressive behavior in BPD. In the context of this model, increased aggressive responses in the mTAP can be explained by a combination of both, deficient impulse control, which is known to correlate positively with aggression in BPD [40, 41], and dysfunctional control of negative affective states such as anger and hostility. Specifically affective dysregulation is regarded as a key aspect in many influential theories of reactive aggression [1, 42, 43], and has been shown to be specifically associated with BPD traits such as resentment and irritation-related aggression [44, 45].

Regarding the assessment of neuropsychological functioning, our data approve former clinical and neuropsychological findings in BPD. Our patient group revealed a lower number of years of education, which was most likely due to the fact that borderline symptoms are associated with specific negative outcomes like lower educational attainment [46]. Furthermore, Haaland and colleagues (2009) found a selective deficit in executive functioning in BPD patients [47], which is also reflected in our results. The aggression and impulsivity questionnaires, including the AQ, RPQ and the BIS-11, confirmed a higher level of aggression and impulsivity in BPD patients, which corroborates previous studies identifying increased levels of aggression and impulsivity as core symptoms of BPD [11, 16].

In conclusion, our results indicate that BPD patients differ behaviourally from healthy controls, with noticeably more aggressive response to provocation in the mTAP. These findings might be highly relevant for all psychiatrists working with BPD patients, because we provide pilot data on a new task that allows psychiatrists to quantify reactive aggression in BPD. This should help to improve diagnostic specificity in two ways: first, it enables clinicians to obtain a clear-cut differentiation between BPD patients and healthy subjects with respect to reactive aggression, and second it should help to clarify which BPD patient might be particularly prone to increased aggression in response to provoking situations during every-day life. Subsequently, this improved diagnostic information regarding aggressive tendencies can be used to support the optimisation and specification of pharmacological and cognitive-behavioural treatment concepts designed to ameliorate aggression.

However, it should be noted that the present study suffers from several limitations: we included only female participants, and the sample size was relatively small. Additionally, the provocation employed in the mTAP is monetary, and this might not tap into the interpersonal aspects that are central to BPD (e.g., sensitivity to rejection and abandonment) and to theoretical and clinical conceptualizations of aggression in BPD. However, since we are not aware of any other behavioural task that might circumvent this limitation and access aggression in a more realistic way that is specifically relevant to BPD patients, we still think that the mTAP provides the best choice for the purpose of our study. Future studies should aim at developing new behavioural paradigms, which focus more on inducing aggressive response via processes that are specific for certain psychiatric conditions, such as BPD. Nevertheless, we provide important pilot data, encouraging future studies to test whether the results and conclusions presented here can be replicated in larger samples, and whether similar results can be obtained when using other aggression-inducing laboratory tasks.
